# Comparison of an AI-based mobile pupillometry system and NPi-200 for pupillary light reflex and correlation with glaucoma-related markers

**DOI:** 10.3389/fneur.2024.1426205

**Published:** 2025-01-09

**Authors:** Damian Jaworski, Karolina Suwała, Bartlomiej J Kaluzny, Jakub J. Kaluzny

**Affiliations:** ^1^Division of Ophthalmology and Optometry, Department of Ophthalmology, Collegium Medicum, Nicolaus Copernicus University, ul. Kornela Ujejskiego, Bydgoszcz, Poland; ^2^Oftalmika Eye Hospital, Bydgoszcz, Poland; ^3^Department of Sensory Organ Studies, Collegium Medicum, Nicolaus Copernicus University, Bydgoszcz, Poland

**Keywords:** artificial intelligence – AI, pupillary light reflex (PLR), glaucoma, software, pupillometry

## Abstract

**Introduction:**

Glaucoma is a leading cause of blindness, often progressing asymptomatically until significant vision loss occurs. Early detection is crucial for preventing irreversible damage. The pupillary light reflex (PLR) has proven useful in glaucoma diagnosis, and mobile technologies like the AI-based smartphone pupillometer (AI Pupillometer) offer a promising solution for accessible screening. This study assesses the reliability of the AI Pupillometer in detecting glaucoma.

**Methods:**

In Experiment 1, 20 healthy participants were assessed using both the AI Pupillometer and the NPi-200 device to evaluate equivalence in measuring PLR. Each eye underwent three trials. Experiment 2 included 46 participants, 24 with primary open-angle glaucoma (POAG) and 22 healthy controls. PLR measurements from the AI Pupillometer were correlated with structural and functional ocular parameters. An additional study expanded the sample to 387 participants (103 glaucoma patients, 284 controls), focusing on differential pupillometry parameters to minimize ambient light interference.

**Results:**

In Experiment 1, the AI Pupillometer demonstrated strong correlations with the NPi-200 in key parameters like initial pupil size (*r* = 0.700), constricted pupil size (*r* = 0.755), and constriction velocity (*r* = 0.541), confirming its reliability. In Experiment 2, although no statistically significant differences in light-corrected PLR parameters were found between groups, glaucoma patients had a marginally higher constricted pupil size (*p* = 0.1632). Significant correlations were observed between pupillometry and advanced ocular imaging results, notably between constriction amplitude and visual field loss. The additional study revealed significant differences in constriction amplitude (*p* = 0.014) and relative pupil size change (*p* = 0.0072) between glaucoma patients and controls, reinforcing the AI Pupillometer’s diagnostic potential.

**Conclusion:**

This study confirms the AI Pupillometer as a reliable, accessible tool for glaucoma screening. Mobile diagnostics could enhance early detection, improving outcomes for glaucoma patients.

## Introduction

Glaucoma stands as the primary cause of irreversible blindness globally, primarily due to the degeneration of the optic nerve. As of 2020, it is estimated that about 76 million suffered from glaucoma, and this figure is expected to continue rising in the years ahead (1). While glaucoma can sometimes manifest with eye pain, in the most cases it presents as a painless condition, characterized by a gradual degeneration of the optic nerve. Regular screening with eye exams is essential for early detection of glaucoma, particularly in its presymptomatic stages. These screenings typically include a standard ophthalmic exam consisting of a fundus examination, measurement of intraocular pressure (IOP), standard automated perimetry (SAT), and more detailed assessments such as optical coherence tomography (OCT) used to evaluate the Retinal Nerve Fiber Layer (RNFL) and the Ganglion Cell Complex (GCC), along with OCT angiography (OCTA) and microperimetry. All these tests have been validated to correlate with the progression of glaucoma, and are helpful in diagnosis, prognosis, and treatment monitoring (2, 3).

Another diagnostic tool, the pupillary light reflex (PLR), measures the diameter of the pupil and its responsiveness to light exposure. This reflex is triggered by retinal ganglion cells, which receive input from photoreceptors via bipolar and amacrine cells (4). Studies to date suggest that the pupillary response is altered in glaucomatous eyes, showing a correlation with RNFL and retinal ganglion cell loss, as well as changes in the visual field (4–8).

The rapid advancement of technology, including artificial intelligence (AI), has significantly influenced medical diagnostics. Mobile PLR has the potential to broaden the availability of glaucoma screening tools, especially for individuals without easy access to an ophthalmologist (9). Such an approach could be essential in facilitating early diagnosis and preventing blindness related to glaucoma.

Given the growing interest in mobile PLR technologies, our study aims to determine whether a conventional infra-red pupillometer (NPi-200, NeurOptics, Inc.) and a smartphone-based pupillometer (AI-based mobile pupillometry system for pupillary light reflex, Solvemed Inc.) (9) produce comparable results (2). Additionally, we evaluated the diagnostic potential of the AI Pupillometer in glaucoma and correlated the pupillometry results obtained with AI Pupillometer with various retinal structural imaging techniques, including OCT and OCTA, and functional test – microperimetry.

## Materials and methods

The study was conducted after obtaining the approval of the Bioethical Committee at the Collegium Medicum in Bydgoszcz, Nicolaus Copernicus University in Torun (approval number KB 427/2021). All conducted procedures in research involving human subjects adhered to the ethical norms of the institutional and/or national research committee, in line with the 1964 Helsinki Declaration and its subsequent modifications or equivalent ethical guidelines.

Each subject received an in-depth eye health evaluation, which included tests for refractive errors (Topcon KR-890, Tokyo, Japan), best-corrected visual acuity (BCVA), slit-lamp biomicroscopy evaluations, and intraocular pressure checks (IOP; Icare TAO1 i, Finland Oy, Vantaa, Finland). Additionally, diagnostic imaging was conducted with Optical Coherence Tomography (OCT) – Spectralis (Heidelberg Engineering, Heidelberg, Germany) and OCT Angiography (OCTA) – Avanti RTVue XR (Optovue, Inc., Fremont, CA, United States) along with macular function testing through the macular analyzer integrity assessment (MAIA) microperimetry (MP) (Centervue, Padova, Italy). All diagnostic procedures were carried out on the same day.

### Comparative study of pupillometry devices: NPi-200 (NeurOptics) and AI Pupillometer (Solvemed)

To evaluate the equivalence of the AI-based mobile pupillometry system for pupillary light reflex (PLR) from Solvemed Inc. (United States), hereafter referred to as the ‘AI Pupillometer,’ and the NeurOptics NPi-200 (NeurOptics, Inc., United States) in a cohort of ophthalmological patients. The study included 20 healthy subjects from Oftalmika Eye Hospital, with assessments conducted sequentially on both the right and left eyes. During the measurements, subjects were instructed to gaze at a distant dark wall. The evaluation commenced with the measurement of the PLR in the right eye using the AI Pupillometer app, while the contralateral eye remained open and uncovered. Following a 20-s interval, the PLR was then measured in the same eye utilizing the NPi-200 pupillometer, with the contralateral eye again kept open and uncovered. This procedure was replicated thrice for each eye under examination.

### Investigation of pupillary light reflex using the smartphone-based AI Pupillometer in glaucoma patients and a control cohort

Experiment 2: the study involved 46 participants, including 24 individuals from the glaucoma group. The inclusion criteria for our study targeted individuals undergoing glaucoma treatment for a period no less than 6 months. To qualify, participants needed a confirmed diagnosis of primary open-angle glaucoma (POAG), characterized by specific conditions: evidence of glaucomatous damage to the optic nerve, a decline in peripapillary Retinal Nerve Fiber Layer (pRNFL) thickness, and/or visual field (VF) deterioration, as identified through Standard Automated Perimetry (SAP). Additionally, all subjects were required to exhibit a normal anterior chamber and open angle, as confirmed by slit-lamp biomicroscopy and gonioscopy, respectively. In glaucoma group, mean Visual Field Index (VFI) was 88.07% with standard deviation 18.12.

As control group, 22 age-matched subjects without any ocular or neurological conditions were selected.

All glaucoma patients were undergoing optimal treatment for glaucoma at the time of the study, ensuring that the disease was managed according to current medical standards. Furthermore, participants had clear optical media, eliminating potential confounders related to ocular transparency. It was also ensured that the patients did not have significant retinal or optic disk changes other than glaucomatous, which could influence the outcome of the study. High-quality imaging studies were utilized for analysis, ensuring that the data collected was of the utmost reliability.

Demographic characteristics are presented of control and glaucoma patient groups are presented in Table 1.

**Table 1:** Demographic data and clinical characteristics of control and glaucoma patient groups.

Characteristic	Control group	Glaucoma group	*P-*value
Age (years)	65.32 ± 4.94 SD	70 ± 6.78 SD	0.0101
Gender	Male: 15 (68.18%)	Male: 12 (50%)	0.3414
	Female: 7 (31.82%)	Female: 12 (50%)	
Intraocular pressure	18.45 ± 2.59SD	16.75 ± 4.75 SD	0.03645
BCVA, EDTRS letters	81.05 ± 6.37 SD	81.75 ± 6.23 SD	0.5963

### Additional study

In this expanded study, we evaluated a larger cohort to further assess the diagnostic utility of the AI Pupillometer in glaucoma. A total of 387 participants were included: 103 patients in the glaucoma group (GG) and 284 in the control group (HC). The study focused on individuals aged 60 years or older, and the groups were age-matched using the Mann–Whitney *U* test (*p* = 0.054). Pupillometry parameters, including Maximally Constricted Pupil Size, Initial Pupil Size, and Maximum Constriction Velocity, were measured and compared between the groups. To account for potential confounding effects of ambient light variation, we also calculated differential parameters, including Constriction Amplitude and Relative Change in Pupil Size. Statistical significance was assessed using the Mann–Whitney *U* test.

### Optical coherence tomography

In our study, patients were subjected to OCT imaging to assess the thickness of pRNFL and GCC. Using a Spectralis, we measured the pRNFL thickness globally with each circular scan aligned with the Optic Nerve Head (ONH). Additionally, the GCC’s thickness was gauged using an Avanti RTVue XR’s glaucoma module, focusing on a macular area 6 mm in diameter. All OCT scans met the OSCAR-IB quality criteria, ensuring consistent and high-quality imaging data for analysis (10).

### Optical coherence tomography angiography

Our study utilized OCTA imaging with an AngioVue Avanti RTVue XR employing the split-spectrum amplitude-decorrelation angiography the split-spectrum amplitude-decorrelation angiography algorithm for detailed retinal vascular visualization. We analyzed the OCTA images as a structural marker, focusing on the microvessel network density through segmentation, not blood flow. The device’s high scan speed and resolution, coupled with DualTrac Motion Correction and 3D PAR in the AngioVue software, ensured artifact-free images. Vessel density was quantitatively assessed from grayscale to binary images, considering both the superficial (SVRP) and deep (DVRP) vascular plexuses, as well as the peripapillary radial peripapillary capillary layer. Only high-quality images, free from motion artifacts were included for analysis.

### Microperimetry

**Figure 1:**
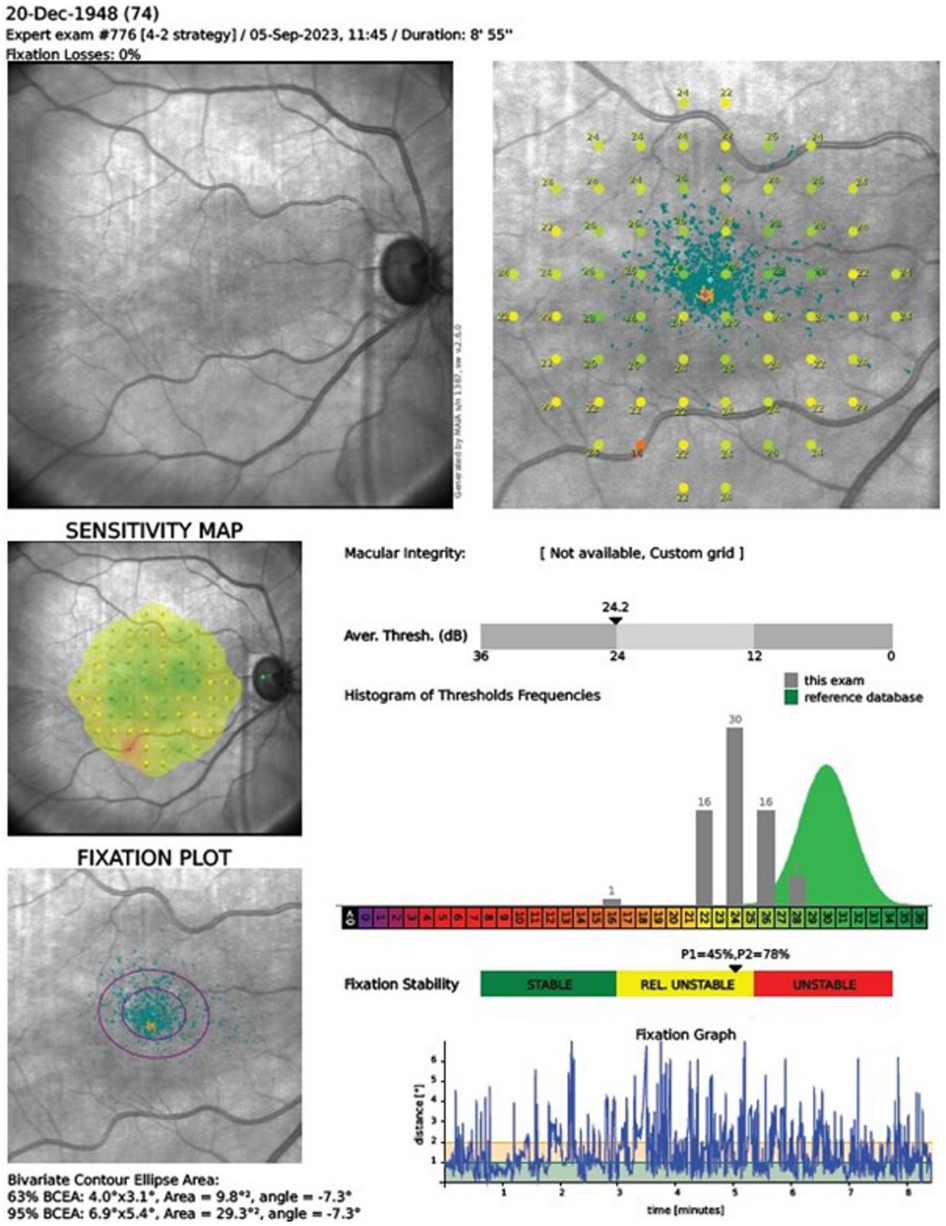
Microperimetry assessment r. The clinical interpretation of MAIA outcomes relies on the manufacturer’s printout, detailing pointwise and average retinal sensitivity across test locations. Sensitivity indices AT and Macular Integrity are depicted in a color chart (green, yellow, red for normal, suspect, abnormal sensitivity, respectively). The printout includes a histogram comparing retinal sensitivity to a normative Gaussian distribution for ages 20–80. It also displays a fixation plot with values for BCEA63 and BCEA95, a fixation stability scale (green, yellow, and red), and a test duration graph.

Our research incorporated MAIA MP system, an advanced integration of Scanning Laser Ophthalmoscopy (SLO), static perimetry, and fundus imaging, for comprehensive microperimetry assessments. This system relies on an infrared superluminescent diode, allowing for the capture of high-resolution images across a spectrum of light attenuation, enhancing the clarity of retinal examinations. With its ability to adjust light intensity in precise increments, the MAIA MP measures retinal threshold sensitivity and examines the stability of fixation. During testing, subjects’ responses to Goldmann-type stimuli are recorded, with an eye-tracker monitoring their gaze in real time to ensure accurate fixation and alignment. The system evaluates fixation stability using two quantitative approaches: calculating the percentage of fixation points within concentric circles of specified radii (notably P1 as 1° and P2 as 2°), and by delineating the bivariate contour ellipse area (BCEA), including 95 and 63% of fixation points (BCEA95 and BCEA63), which reflects the spatial distribution of eye fixation. The clinical interpretation of these findings is facilitated by the MAIA’s analytical printout, as showcased in Figure 1. This report details pointwise retinal sensitivity and the average sensitivity across all test loci, color-coded to signify normal, suspect, or abnormal levels. Additionally, the system provides a histogram of threshold frequencies, allowing for statistical comparison to normative data, along with a fixation plot that visually and numerically represents fixation stability based on BCEA and P1, P2 metrics. This comprehensive dataset is essential for evaluating the integrity of macular function.

### Analysis and statistics

We conducted various statistical analyses using Python 3.11, utilizing the *SciPy* library for hypothesis testing—including independent *t*-tests to assess differences in demographics and clinical characteristics between the glaucoma and control groups, Pearson correlations, and Mann–Whitney *U* tests—and NumPy for array manipulation and mathematical operations. For linear regression analyses and to calculate regression coefficients, we employed the *statsmodels* library, which provided comprehensive methods to model and analyze the relationship between variables. To ensure our data met the assumptions necessary for the validity of our statistical tests, we assessed the normality using the Kolmogorov–Smirnov test within the *SciPy* library. A *p*-value of less than 0.05 was considered statistically significant.

### Pupillometry

The Solvemed AI Pupillometer is a smartphone-based software as medical device (SaMD), capable of measuring and tracking pupil size and reactivity without the need for any additional hardware. The device uses smartphone camera and deep learning to produce pupillometric parameters describing the pupil light reflex. Notably, the device captures the level of ambient light during measurements and corrects for any excessive or deficient lighting level to produce clinically meaningful results (9, 11). The NeurOptics NPi-200 pupillometer stands as a specialized instrument to measure the PLR. This device employs an infrared camera system capable of capturing images of the pupil under various lighting conditions, thereby ensuring consistent measurement accuracy. Unlike conventional methods that may be influenced by ambient light, the NPi-200’s technology allows for undisturbed observation of the pupil’s response to light stimulation. The device’s operation does not require dimming of lights or specific environmental adjustments. Furthermore, the NPi-200 generates comprehensive reports that detail the dynamics of the pupillary response. Recent advances in smartphone-based pupillometry have enabled applications in challenging settings, where the AI Pupillometer’s software effectively stabilizes measurements in dynamic environments, reducing motion artifacts and enhancing sensitivity to pupil dynamics.

## Results

### Comparative analysis of retinal parameters in control and glaucoma groups

The statistical analysis revealed a significant differences between the control group and the glaucoma group for the following parameters: pRNFL thickness (*p* < 0.0001), whole image SRVD (*p* = 0.0082), perifoveal SRVD (*p* = 0.004), average GCC (*p* = 0.0002), and average threshold (AT) in MP (*p* = 0.0093).

Detailed data are presented in Table 2.

**Table 2:** Statistics for significantly different OCT and OCTA parameters for glaucoma and control group.

	Glaucoma		Control		
Parameter name	Mean	Std	Mean	Std	*P*-value
Superficial vascular plexus whole image (%)	44.550	4.464	48.029	3.267	0.0082
Superficial vascular plexus perifovea (%)	44.650	4.815	48.829	3.650	0.0040
GCC average (μm)	84.087	12.339	99.476	11.512	0.0002
Average pRNFL (μm)	73.81	15.81	99.37	12.09	<0.0001
Average sensitivity threshold (dB)	19.000	8.153	24.544	1.962	0.0093

### Correlation analysis between Solvemed AI Pupillometer parameters and advanced ocular imaging techniques

The comparative analysis of the results from the AI Pupillometer pupillary test, both for light-corrected and uncorrected parameters, did not demonstrate statistically significant differences between the glaucoma and control groups. Nevertheless, the value of the ‘Constricted Pupil Size’ light corrected parameter was notably higher in the glaucoma group compared to the control group, with a *p*-value of 0.1632. Detailed data are presented in Tables 3, 4.

**Table 3:** Statistical differences for Solvemed AI Pupillometer light un-corrected parameters for glaucoma vs. control.

	Glaucoma		Control		
Parameter name	Mean	Std	Mean	Std	*P*-value
Initial pupil size (mm)	3.205	0.483	3.096	0.379	0.2696
Constricted pupil size (mm)	2.696	0.515	2.588	0.279	0.2535
Constriction amplitude (mm)	0.509	0.197	0.508	0.249	0.9790
Constriction velocity (mm/s)	1.207	0.542	1.277	0.653	0.6093

**Table 4:** Statistical differences for Solvemed AI Pupillometer light corrected parameters for glaucoma vs. control with parameters as absolute values.

	Glaucoma		Control		
Parameter name	Mean	Std	Mean	Std	*P*-value
Constricted pupil size (mm)	3.970	0.257	3.861	0.411	0.1632
Constriction amplitude (mm)	0.139	0.306	0.138	0.308	0.9847
Constriction Velocity (mm/s)	1.614	1.017	1.684	1.166	0.7792

Furthermore, we performed an analysis to explore the correlations between parameters measured by the Solvemed AI Pupillometer and the average pRNFL and GCC, both separately for glaucoma and control groups and combined for all participants. Table 5 presents the correlations for light-corrected parameters, while Table 6 covers the uncorrected parameters, with each employing Spearman correlation and providing *p*-values in parentheses. This comprehensive analysis found no statistically significant differences or strong correlations within or across the groups.

**Table 5:** Correlation between Solvemed AI Pupillometer light uncorrected parameters and average pRNFL and GCC calculated with Spearman correlation.

Solvemed AI Pupillometer parameter	Glaucoma		Control		All	
	G RNFL	GCC average	G RNFL	GCC average	G RNFL	GCC average
Initial pupil size (mm)	0.0428 (0.881)	0.0149 (0.620)	0.168 (0.607)	0.0466 (0.863)	0.0161 (0.744)	0.0308 (0.628)
Constricted pupil size (mm)	−0.00235 (0.551)	0.132 (0.580)	0.222 (0.479)	0.000505 (0.873)	0.0489 (0.522)	0.0927 (0.297)
Constriction amplitude (mm)	−0.000564 (0.298)	−0.00219 (0.801)	−0.0526 (0.701)	−0.0160 (0.512)	−0.0831 (0.979)	−0.0546 (0.731)
Constriction velocity (mm/s)	−0.00958 (0.107)	−0.0760 (0.679)	0.0520 (0.951)	0.0715 (0.871)	0.00644 (0.236)	−0.0302 (0.336)

*p-values given in the brackets.*

**Table 6:** Correlation between Solvemed AI Pupillometer light corrected parameters and average pRNFL and GCC calculated with Spearman correlation.

Solvemed AI Pupillometer parameter	Glaucoma		Control		All	
	G RNFL	GCC average	G RNFL	GCC average	G RNFL	GCC average
Constricted pupil size (mm)	0.0435 (0.890)	0.0237 (0.652)	0.323 (0.154)	0.186 (0.411)	0.0162 (0.563)	0.0393 (0.472)
Constriction amplitude (mm)	−0.0500 (0.245)	−0.0947 (0.934)	0.0889 (0.586)	0.169 (0.419)	−0.0989 (0.786)	−0.086 (0.751)
Constriction velocity (mm/s)	−0.157 (0.456)	−0.0952 (0.553)	0.105 (0.573)	0.193 (0.195)	−0.00231 (0.999)	0.0292 (0.851)

*P-values given in the brackets.*

The data analysis combining Solvemed AI Pupillometer light-corrected parameters with microperimetry, OCT, and OCTA results, across both study groups, revealed significant correlations with a *p*-value below 0.01, between the following parameters: initial pupil size and fix losses; constricted pupil size and IOP, Area63, Area95, P1, P2; constriction amplitude – light corrected and FAZ, PERIM, superficial fovea, deep fovea, Area63, Area95, P1, P2; constriction velocity – light corrected and Area63, Area95, P1, P2.

When comparing the PLR parameters from the Solvemed AI Pupillometer with parameters from OCT and microperimetry, only correlations with an absolute value of 0.25 and a *p*-value below 0.01 were included to ensure that only statistically significant and moderately strong relationships were considered. Given that 40 correlations were evaluated, the threshold for significance was set at 0.0013 using the Bonferroni correction. This correction adjusts the *p*-value threshold to control for the increased risk of false positives that occurs when multiple comparisons are made. After applying the Bonferroni correction, the significant correlations identified were between constriction amplitude and Area 96, Area 65, and P2. These results suggest specific relationships that are statistically robust even after adjusting for multiple tests. Detailed data are presented in Table 7.

**Table 7:** Correlations between Solvemed AI Pupillometer light corrected parameters and OCT and microperimetry parameters, calculated with Spearman correlation test with *p*-values.

Solvemed AI Pupillometer	OCT and microperimetry	Correlation value
Initial pupil size	Fix losses	0.203 (0.0229)
Initial pupil size	IOP	−0.253 (0.0099)
Constricted pupil size	Area63	0.366 (0.0047)
Constricted pupil size	Area95	0.368 (0.0040)
Constricted pupil size	P1	−0.354 (0.0060)
Constricted pupil size	P2	−0.348 (0.0146)
Constriction amplitude corrected	FAZ	0.315 (0.0087)
Constriction amplitude corrected	PERIM	0.321 (0.0067)
Constriction amplitude corrected	Superficial Fovea	−0.280 (0.019)
Constriction amplitude corrected	Deep fovea	−0.347 (0.0048)
Constriction amplitude corrected	Area63	−0.417 (0.0004)
Constriction amplitude corrected	Area95	−0.414 (0.0003)
Constriction amplitude corrected	P1	0.398 (0.0014)
Constriction amplitude corrected	P2	0.386 (0.0009)
Constriction velocity corrected	Area63	−0.246 (0.0153)
Constriction velocity corrected	Area95	−0.253 (0.0136)
Constriction velocity corrected	P1	0.284 (0.0062)
Constriction velocity corrected	P2	0.230 (0.0293)

### Reliability and correlation analysis of pupillometry parameters: Solvemed AI Pupillometer vs. NeurOptics NPi-200

**Figure 2:**
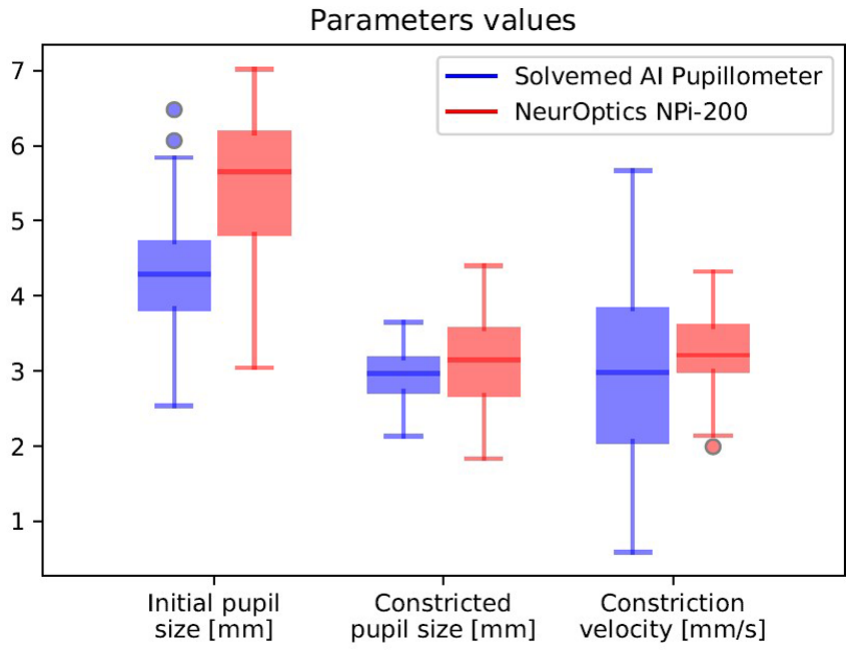
Correlation between the parameters reported by Solvemed AI Pupillometer and NeurOptics NPi-200.

**Figure 3:**
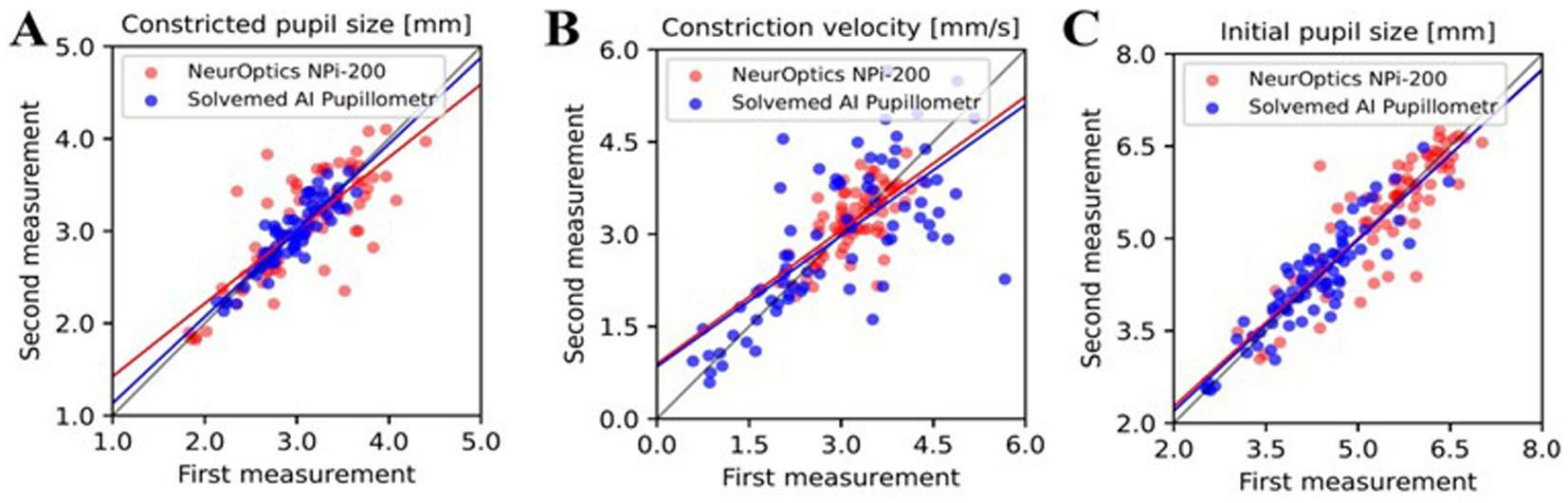
The correlation between parameters reported by the Solvemed AI Pupillometer and the NeurOptics NPi-200 is depicted in graphs **(A–C)**, representing the first and second measurements with both devices for constricted pupil size, constriction velocity, and initial pupil size, respectively.

In the comparative analysis of results obtained from the Solvemed AI Pupillometer and the NeurOptics NPi-200, we conducted a correlation analysis between the parameters reported by both devices. For initial pupil size, constricted pupil size, and constriction velocity, Pearson’s correlation coefficients were estimated to be 0.700, 0.755, and 0.541, respectively, all with a *p*-value of <0.0001. Detailed data are presented in Table 8 and Figure 2. Further analysis revealed a high reliability for the Solvemed AI Pupillometer in comparison to the NeurOptics NPi-200. This reliability was assessed by calculating the differences between the first and second, and second and third measurements. Linear regression was applied, and the resulting coefficients were reported. For the parameters of initial pupil size, constricted pupil size, and constriction velocity, the *R*-values were 0.903, 0.893, and 0.717, respectively, for the Solvemed AI Pupillometer, compared to 0.869, 0.773, and 0.680 for the NPi-200. All these values yielded a *p*-value of <0.0001. Detailed data are presented in Tables 9, 10. The scatterplot graphs illustrating correlations of constricted pupil size, constriction velocity, and initial pupil size are collectively presented in Figures 3A–C, respectively.

**Table 8:** Correlation between the parameters reported by Solvemed AI Pupillometer and NeurOptics NPi-200.

Parameter name	Pearson’s correlation coefficient	Correlation *P*-value
Initial pupil size (mm)	0.700	<0.0001
Constricted pupil size (mm)	0.755	<0.0001
Constriction velocity (mm/s)	0.541	<0.0001

**Table 9:** Reliability of Solvemed AI Pupillometer vs. NeurOptics NPi-200.

	Solvemed AI Pupillometer			NeurOptics NPi-200		
Parameter	Pearson’s correlation coefficient	Linear regression coefficient	*P*-value	Pearson’s correlation coefficient	Linear regression coefficient	*P*-value
Initial pupil size (mm)	0.903	0.926	<0.0001	0.869	0.911	<0.0001
Constricted pupil size (mm)	0.893	0.937	<0.0001	0.773	0.792	<0.0001
Constriction velocity (mm/s)	0.717	0.709	<0.0001	0.680	0.723	<0.0001

Calculated as a difference between the parameters of first vs. second and second vs. third measurement.

**Table 10:** Mean and median differences with standard deviation for paired measurements.

	Solvemed AI Pupillometer			NeurOptics NPi-200		
Parameter	Mean difference	Median difference	Std	Mean difference	Median difference	Std
Initial pupil size (mm)	0.287	0.230	0.219	0.329	0.260	0.339
Constricted pupil size (mm)	0.125	0.101	0.097	0.282	0.280	0.277
Constriction velocity (mm/s)	0.660	0.496	0.621	0.305	0.210	0.271

### Additional study on the application of the AI Pupillometer in glaucoma diagnostics

In an expanded examination of the utility of the AI Pupillometer in glaucoma diagnostics, we conducted a supplementary study encompassing a broader cohort of patients. This investigation aimed to further validate the efficacy of the AI Pupillometer by including a larger sample size, specifically targeting individuals aged 60 years or older. The study comprised 103 patients in the glaucoma group (GG) and 284 individuals in the control group (HC), with age matching performed to ensure comparability between the groups (Mann–Whitney *U* test, *p* = 0.054; GG: 73.18 ± 0.75 years, HC: 71.28 ± 0.39 years, mean ± s.e.m). We evaluated the same pupillometry parameters for both groups, employing the Mann–Whitney *U* Test to determine statistical significance (Table 11). Three previously reported parameters—Maximally Constricted Pupil Size (GG: 3.00 mm ± 0.06, HC: 2.95 mm ± 0.04; *p* = 0.59, mean ± s.e.m), Initial Pupil Size (GG: 3.88 mm ± 0.07, HC: 3.95 mm ± 0.05; *p* = 0.38, mean ± s.e.m), and Maximum Constriction Velocity (GG: 3.49 mm/s ± 0.20, HC: 3.43 mm/s ± 0.12; *p* = 0.89, mean ± s.e.m)—did not significantly differ between the groups.

**Table 11:** Statistical differences between pupillometry parameters in the expanded study on glaucoma and healthy cohorts.

Parameter	Mean (glaucoma group)	Standard error of the mean (glaucoma group)	Mean (control group)	Standard error of the mean (control group)	*p*-value
Age (years)	73.18	0.75	71.28	0.39	0.054
Initial pupil size (mm)	3.88	0.07	3.95	0.05	0.38
Maximally constricted pupil size (mm)	3.00	0.06	2.95	0.04	0.59
Maximum constriction velocity (mm/s)	3.49	0.20	3.43	0.12	0.89
Constriction amplitude (mm)	0.87	0.04	0.99	0.03	0.014
Relative change in pupil size (%)	22.03	0.91	24.45	0.52	0.0072

Given the suspicion that ambient light variation may confound these measurements, we calculated differential parameters utilizing the larger dataset. We determined the Constriction Amplitude as the difference between the initial and maximally constricted pupil sizes (GG: 0.87 mm ± 0.04, HC: 0.99 mm ± 0.03; *p* = 0.014, mean ± s.e.m), and the Relative Change as the difference between the initial and maximally constricted pupil size divided by the initial size (GG: 22.03% ± 0.91, HC: 24.45% ± 0.52; *p* = 0.0072, mean ± s.e.m). This approach revealed significant differences between groups in the Constriction Amplitude and the Relative Change in Pupil Size, suggesting that differential parameters might help reduce the impact of confounding factors, thereby highlighting the distinct characteristics between the groups.

## Discussion

Evaluating PLR has been a recognized method for assessing the integrity of afferent visual pathways, along with the sympathetic and parasympathetic nervous systems. Yet, as noted in a comprehensive study by Hennessy et al. (12), factors like test duration, dependency on the operator, and variability in pupillary reactions to ambient light variations have constrained the use of PLR in glaucoma diagnosis. In another research, Kalaboukhova et al. (13) utilized a specialized, non-commercial pupillometer to measure pupillary area ratio (PAR), contraction velocity ratio, and dilation velocity ratio (PDVR). Their findings indicated notable disparities in PAR and PDVR between patients with glaucoma and those in the control group.

At the moment, two techniques are used to evaluate the PLR: digital infrared pupillometry and a more traditional visual inspection of pupils, for example with a penlight, which is commonly used in clinical practice. However, reliance on subjective assessment leads to notable imprecision, with an average error margin of approximately 0.5 mm, significantly higher than that of its digital counterpart (14–17). In comparison, digital technologies have demonstrated superior accuracy, reducing the median error to around 0.36 mm (18).

Typically, pupillary reactivity is described as ‘normal,’ ‘sluggish,’ or ‘fixed,’ which are often inconsistent across different examiners, what was shown in the study where they compared assessments between examiners and revealed only moderate agreement in evaluations of pupil size and reactivity (*κ* values of 0.54 and 0.40, respectively). Notably, a considerable discrepancy was observed in the identification of non-reactive pupils, a critical indicator in severe traumatic brain injuries, with only a third of cases identified manually aligning with digital assessments (19). A separate study highlighted an 18% discordance in PLR assessments between healthcare professionals and digital pupillometry, showing the limitations of subjective evaluation (19).

On the other hand, digital infrared pupillometry, now considered the clinical gold standard, employs a device equipped with an LED light and an infrared camera. This device utilizes infrared technology to delineate the pupil’s boundary and then applies an LED flash to prompt and monitor pupillary constriction, enabling the calculation of a Neurological Pupil Index score. This method has a relatively lower median error rate of approximately 0.23 mm (15). For context, the average resting pupil diameter in a healthy individual is roughly 3.4 mm, which narrows by about 0.88 mm following light exposure of 180–200 cd/m (15). While digital pupillometry demonstrates high reliability in standard clinical usage, its broader implementation is hindered by several drawbacks, particularly in high costs and requires single-use components per patient.

In our study, we first aimed to compare the results of pupillary light reflex measurements obtained using two different devices: a standard medical device, NeurOptics NPi-200, and another device based on an iOS mobile application – the Solvemed AI Pupillometer. We assessed the reliability by comparing the repeatability of measurements between the first and second, and second and third measurements. Furthermore, to evaluate the new device’s utility in glaucoma diagnostics, we compared the pupillary light reflex (PLR) between a control group and patients with glaucoma. In Experiment 1, which focused on comparing the AI Pupillometer with the NPi-200 device, the use of a mobile application-based device that provides light-corrected pupil reaction measurements emerged as a promising direction due to its portability and accessibility through mobile phone app installation. Our findings, validated through Pearson correlation, revealed a strong correlation between measurements taken by both devices for initial pupil size (0.700), constricted pupil size (0.755), and a moderate correlation for constriction velocity (0.541). Additionally, the analysis demonstrated high reliability for both devices across all three PLR parameters, with the R value being higher using the Solvemed app for all aforementioned parameters.

Previous research on pupillography has validated its utility in preoperative pupil assessments for refractive procedures such as laser vision correction or intraocular lens implantation. Additionally, it facilitates an objective evaluation of the visual pathway function, including the retina, in conditions like retinal vein occlusion and glaucoma (7, 20). Changes in the pupillary light reflex have also been studied in neurological diseases such as epilepsy, Alzheimer’s disease, Parkinson’s disease, as well as in acute brain injuries and concussions (21–25).

Recent research on the application of pupillometry has shown a growing interest in the use of mobile devices like smartphones. This type of pupillometry has been studied for various applications, including in traumatic brain injury, for the detection of acute large vessel occlusion, and for At-Home Pupillometry using Smartphone Facial Identification Cameras (26–28).

The results of our study confirms findings from other research on the differences in the SVRP, GCC, and retinal sensitivity assessed through microperimetry between patients with glaucoma and healthy individuals (2, 3). Comparative analysis of PLR parameters with imaging and functional test results for the same patient group revealed a correlation of several parameters from the conducted studies with selected PLR metrics. There was no statistically significant correlation between PLR parameters and glaucomatous damage markers, such as GCC, SRVP, and AT.

In Experiment 2, which assessed the AI Pupillometer in both glaucoma patients and controls, certain PLR parameters showed correlations with glaucoma-related structural and functional markers. Similar correlations have been identified in previous studies. For example, a 2022 study by Zabel et al. (2) demonstrated correlations between parameters such as SRVP, P1, P2, BCEA65, and BCEA95 with typical glaucoma markers like GCC, RNFL, and glaucoma progression, while no significant correlation was found for DRVP. Other studies have shown correlations between FAZ, PERIM, and DRVP, with the occurrence of glaucoma (29–31).

There are no significant correlations between the studied parameters and PLR for both the glaucoma and control groups, analyzed separately, which can be explained by the relatively high VFI index in the glaucoma group, indicating that a large part of the patients with glaucoma have preperimetric glaucoma and small study group in primary study. We can hypothesize that the lack of sensitivity changes in the central part of the visual field at the initial stages of glaucoma development may not cause disturbances in PLR parameters. In published studies on glaucoma, in many cases, glaucoma groups are differentiated into subgroups with preperimetric and perimetric glaucoma, with the aim of obtaining more accurate results (32, 33). However, in the additional study conducted on a broader cohort of patients with glaucoma, compared with healthy subjects, statistically significant differences were found in PLR parameters such as constriction amplitude and relative change in pupil size. These differences might be explained by the broader cohort.

Our study had several limitations. Firstly, the small patient cohort in primary study and in the additional study, the structural and functional parameters of the retina and optic nerve of the subjects were not included. Secondly, the absence of patient selection based on the use of anti-glaucoma and systemic medications, which could affect the pupillary light reflex. Thirdly, the lack of patient selection based on their cataract surgery status, whether postoperative or preoperative. Another limitation of this study was that the order of testing was not fixed, with the App always being tested first. While we cannot rule out order effects, previous data suggest that the PLR adaptation is a small effect and are therefore unlikely to affect the results (33). Moreover, our analyses involve correlation, which means any consistent adaptation or bias should not alter the result.

The findings from Experiment 1, comparing the AI Pupillometer with traditional pupillometry devices, and Experiment 2, investigating its use in glaucoma and control groups, align with previous research and offer promising prospects for future studies on mobile PLR technology in glaucoma assessment. Additionally, our study highlights a previously underexplored relationship between PLR and parameters such as retinal vessel density and gaze fixation stability. The comparative study of the mobile device –AI-Pupillometer, against the gold standard pupillometer – NPi-200 confirms its non-inferiority, and even slightly better performance. The correlation with structural and functional test parameters suggests the potential applicability of PLR in examining patients with glaucoma. Our findings align with previous research demonstrating the diagnostic value of the PLR in glaucoma detection. To further support this, Suo et al. (34) conducted a meta-analysis that evaluated the sensitivity and specificity of computerized pupillary assessments for glaucoma detection. Their results showed that PLR is both a sensitive and specific method for detecting glaucomatous damage, providing strong validation for its use in clinical practice. Nonetheless, further research on a larger patient cohort is necessary to confirm these findings.

## Data Availability

The raw data supporting the conclusions of this article will be made available by the authors, without undue reservation.

## References

[1] Allison K Patel D Alabi O. Epidemiology of Glaucoma: the past, present, and predictions for the future. Cureus. (2020) 12:e11686. doi: 10.7759/CUREUS.11686, PMID: 33391921 PMC7769798

[2] Zabel K Zabel P Suwala K Gorczyca A Jaworski D Kaluzna M. Alterations in fixation indices in primary open-angle glaucoma by microperimetry. J Clin Med. (2022) 11:2368. doi: 10.3390/JCM11092368, PMID: 35566493 PMC9102428

[3] Scuderi L Gattazzo I de Paula A Iodice CM Di Tizio F Perdicchi A. Understanding the role of microperimetry in glaucoma. Int Ophthalmol. (2022) 42:2289–301. doi: 10.1007/S10792-021-02203-3, PMID: 35094226

[4] Chang DS Arora K Boland MV Friedman DS. The relationship between quantitative pupillometry and estimated ganglion cell counts in patients with Glaucoma. J Glaucoma. (2019) 28:238–42. doi: 10.1097/IJG.0000000000001183, PMID: 30624390

[5] Bayraktar S Hondur G Şekeroǧlu MA Şen E. Evaluation of static and dynamic pupillary functions in early-stage primary open angle Glaucoma. J Glaucoma. (2023) 32:E90–4. doi: 10.1097/IJG.0000000000002212, PMID: 36971579

[6] Quan Y Duan H Zhan Z Shen Y Lin R Liu T. Binocular head-mounted chromatic pupillometry can detect structural and functional loss in glaucoma. Front Neurosci. (2023) 17:7619. doi: 10.3389/FNINS.2023.1187619, PMID: 37456990 PMC10346847

[7] Quan Y Duan H Zhan Z Shen Y Lin R Liu T. Evaluation of the glaucomatous macular damage by chromatic pupillometry. Ophthalmol Ther. (2023) 12:2133–56. doi: 10.1007/S40123-023-00738-5, PMID: 37284935 PMC10287851

[8] Chang DS Boland MV Arora KS Supakontanasan W Chen BB Friedman DS. Symmetry of the pupillary light reflex and its relationship to retinal nerve Fiber layer thickness and visual field defect. Invest Ophthalmol Vis Sci. (2013) 54:5596–601. doi: 10.1167/IOVS.13-12142, PMID: 23860751 PMC4591738

[9] Bogucki A John I Zinkiewicz Ł Jachura M Jaworski D Suwała K. Machine learning approach for ambient-light-corrected parameters and the pupil reactivity score in smartphone-based pupillometry. Front Neurol. (2024) 15:1363190. doi: 10.3389/fneur.2024.1363190, PMID: 38654735 PMC11037402

[10] Tewarie P Balk L Costello F Green A Martin R Schippling S. The OSCAR-IB consensus criteria for retinal OCT quality assessment. PLoS One. (2012) 7:e34823. doi: 10.1371/JOURNAL.PONE.0034823, PMID: 22536333 PMC3334941

[11] John I Yari Z Bogucki A Swiatek M Chrost H Wlodarski M. Unsupervised deep learning-driven stabilization of smartphone-based quantitative pupillometry for mobile emergency medicine. Proc Int Symp Biomed Imaging. (2024). doi: 10.1109/ISBI56570.2024.10635305

[12] Hennessy AL Katz J Ramakrishnan R Krishnadas R Thulasiraj RD Tielsch JM. The utility of relative afferent pupillary defect as a screening tool for glaucoma: prospective examination of a large population-based study in a south Indian population. Br J Ophthalmol. (2011) 95:1203–6. doi: 10.1136/BJO.2010.194217, PMID: 21349935

[13] Kalaboukhova L Fridhammar V Lindblom B. Relative afferent pupillary defect in glaucoma: a pupillometric study. Acta Ophthalmol Scand. (2007) 85:519–25. doi: 10.1111/J.1600-0420.2006.00863.X, PMID: 17573859

[14] Ong C Hutch M Smirnakis S. The effect of ambient light conditions on quantitative pupillometry. Neurocrit Care. (2019) 30:316–21. doi: 10.1007/S12028-018-0607-8, PMID: 30218349

[15] Prabhakaran K Petrone P Lombardo G Stoller C Policastro A Marini CP. Mortality rates of severe traumatic brain injury patients: impact of direct versus non-direct transfers. J Surg Res. (2017) 219:66–71. doi: 10.1016/J.JSS.2017.05.103, PMID: 29078912

[16] Olson DWM Stutzman S Saju C Wilson M Zhao W Aiyagari V. Interrater reliability of pupillary assessments. Neurocrit Care. (2016) 24:251–7. doi: 10.1007/S12028-015-0182-1 26381281

[17] Papageorgiou E Ticini LF Hardiess G Schaeffel F Wiethoelter H Mallot HA. The pupillary light reflex pathway: cytoarchitectonic probabilistic maps in hemianopic patients. Neurology. (2008) 70:956–63. doi: 10.1212/01.WNL.0000305962.93520.ED, PMID: 18347318

[18] Mariakakis A Baudin J Whitmire E Mehta V Banks MA Law A. PupilScreen. Proc ACM Interact Mobile Wear Ubiquitous Technol. (2017) 1:1–27. doi: 10.1145/3131896, PMID: 39076787

[19] Olson DM Stutzman SE Atem F Kincaide JD Ho TT Carlisle BA. Establishing normative data for Pupillometer assessment in neuroscience intensive care: the “END-PANIC”. Registry J Neurosci Nurs. (2017) 49:251–4. doi: 10.1097/JNN.0000000000000296, PMID: 28661950

[20] Turk HB Bitirgen G Satirtav G Kerimoglu H. Assessment of pupillary light reflex using dynamic pupillometry in laser-treated eyes with retinal vein occlusion. Eur J Ophthalmol. (2021) 31:2505–10. doi: 10.1177/1120672120969038, PMID: 33118385

[21] You S Hong JH Yoo J. Analysis of pupillometer results according to disease stage in patients with Parkinson’s disease. Sci Rep. (2021) 11:17880. doi: 10.1038/S41598-021-97599-4, PMID: 34504251 PMC8429555

[22] Carrick FR Azzolino SF Hunfalvay M Pagnacco G Oggero E D’arcy RCN. The pupillary light reflex as a biomarker of concussion. Life. (2021) 11:1104. doi: 10.3390/LIFE11101104, PMID: 34685475 PMC8537991

[23] El Ahmadieh TY Bedros N Stutzman SE Nyancho D Venkatachalam AM MacAllister M. Automated pupillometry as a triage and assessment tool in patients with traumatic brain injury. World Neurosurg. (2021) 145:e163–9. doi: 10.1016/J.WNEU.2020.09.152, PMID: 33011358

[24] Chougule PS Najjar RP Finkelstein MT Kandiah N Milea D. Light-induced pupillary responses in Alzheimer’s disease. Front Neurol. (2019) 10:360. doi: 10.3389/FNEUR.2019.00360, PMID: 31031692 PMC6473037

[25] Godau J Bierwirth C Rösche J Bösel J. Quantitative infrared pupillometry in non-convulsive status epilepticus. Neurocrit Care. (2021) 35:113–20. doi: 10.1007/S12028-020-01149-1, PMID: 33215395

[26] Barry C De Souza J Xuan Y Holden J Granholm E Wang EJ. At-home pupillometry using smartphone facial identification cameras. Proc SIGCHI Conf Hum Fact Comput Syst CHI Conf. (2022) 2022:2493. doi: 10.1145/3491102.3502493, PMID: 38031623 PMC10686294

[27] Maxin AJ Gulek BG Chae J Winston G Weisbeek P McGrath LB. A smartphone pupillometry tool for detection of acute large vessel occlusion. J Stroke Cerebrovasc Dis. (2023) 32:107430. doi: 10.1016/J.JSTROKECEREBROVASDIS.2023.107430, PMID: 37857150

[28] Maxin AJ Gulek BG Lee C Lim D Mariakakis A Levitt MR. Validation of a smartphone pupillometry application in diagnosing severe traumatic brain injury. J Neurotrauma. (2023) 40:2118–25. doi: 10.1089/NEU.2022.0516, PMID: 37464770

[29] Nishida T Moghimi S Walker E Gunasegaran G Wu J-H Kamalipour A. Association of foveal avascular zone change and glaucoma progression. Br J Ophthalmol. (2023) 108:1101–6. doi: 10.1136/BJO-2023-323970, PMID: 38164585 PMC11192860

[30] Choi J Kwon J Shin JW Lee J Lee S Kook MS. Quantitative optical coherence tomography angiography of macular vascular structure and foveal avascular zone in glaucoma. PLoS One. (2017) 12:e0184948. doi: 10.1371/JOURNAL.PONE.0184948, PMID: 28934255 PMC5608222

[31] Li F Lin F Gao K Cheng W Song Y Liu Y. Association of foveal avascular zone area with structural and functional progression in glaucoma patients. Br J Ophthalmol. (2022) 106:1245–51. doi: 10.1136/BJOPHTHALMOL-2020-318065, PMID: 33827858

[32] Preiser D Lagrèze WA Bach M Poloschek CM. Photopic negative response versus pattern electroretinogram in early glaucoma. Invest Ophthalmol Vis Sci. (2013) 54:1182–91. doi: 10.1167/IOVS.12-11201, PMID: 23307968

[33] Horn FK Kremers J Mardin CY Jünemann AG Adler W Tornow RP. Flicker-defined form perimetry in glaucoma patients. Graefes Arch Clin Exp Ophthalmol. (2015) 253:447–55. doi: 10.1007/S00417-014-2887-9, PMID: 25511293

[34] Suo L Zhang D Qin X Li A Zhang C Wang Y. Evaluating state-of-the-art computerized pupillary assessments for Glaucoma detection: a systematic review and meta-analysis. Front Neurol. (2020) 11:552573. doi: 10.3389/FNEUR.2020.00777/BIBTEX PMC7403439 32849229

